# ARTAG in the population‐based Vantaa 85+ Study of the oldest old

**DOI:** 10.1002/alz70861_108529

**Published:** 2025-12-23

**Authors:** Benjamin Englert, Ella Ahvenainen, Sara Emilia Savola, Kia Colangelo, Tuomo Polvikoski, Liisa Myllykangas

**Affiliations:** ^1^ University of Helsinki, Helsinki Finland; ^2^ HUS Diagnostic Center, Helsinki Finland; ^3^ Helsinki University Hospital, Helsinki Finland; ^4^ Newcastle University, Newcastle upon Tyne UK

## Abstract

**Background:**

Aging‐ and disease‐associated neuropathological features are frequently found in brains of the elderly, with a plethora of possible combinations of neurodegenerative pathologies. This holds especially true for the heterogeneity of pathologies underlying cognitive decline in the oldest old population, where the role of concomitant pathologies such as cerebrovascular disease, hippocampal sclerosis of aging (HS‐A) or limbic‐predominant age‐related TDP‐43 encephalopathy‐neuropathological changes (LATE‐NC) is pronounced. One condition found in the aging brain that often appears together with other neurodegenerative pathologies is aging‐related tau astrogliopathy (ARTAG). ARTAG refers to astrocytic accumulation of abnormally phosphorylated tau protein (pTau). ARTAG consists of thorn‐shaped astrocytes (TSA) and granular/fuzzy astrocytes (GFA) as characteristic cytomorphological features in various sublocations, such as white (WM) and grey (GM) matter, subependymal, subpial and perivascular. Studies addressing the detailed impact of various ARTAG subtypes in population‐based cohorts are sparse. This data would be needed to understand the complex interdependency of neurodegenerative changes in the very elderly.

**Method:**

The population‐based Vantaa 85+ Study cohort contains 304 brains of subjects older than 85 years. Samples of the amygdala, hippocampus, basal temporal cortex, frontal and occipital lobe, and midbrain were stained with antibodies against pTau. The extent of ARTAG was evaluated according to previously described criteria based on light microscopy analysis. Alzheimer disease associated pathologies, CAA, HS‐A, LATE‐NC and cerebrovascular alterations were previously determined in this cohort. The results presented were controlled for age at death, sex and Braak stage.

**Result:**

HS‐A showed an association with hippocampal (*p* =0.001) and basotemporal (*p* =0.024) WM ARTAG and with hippocampal (*p* =0.033) and basotemporal (*p* =0.028) TSA. LATE‐NC and hippocampal WM ARTAG (*p* =0.032) and any basotemporal ARTAG (*p* =0.011) were associated. Capillary (type 1) cerebral amyloid angiopathy was associated with WM (*p* =0.004) and subependymal (*p* =0.048) TSA in the medial temporal lobe (MTL), whereas frontal ARTAG (*p* =0.024) and MTL TSA (*p* =0.008) were associated with the presence of cerebral microinfarcts.

**Conclusion:**

In the oldest old, ARTAG is linked to various copathologies, like LATE‐NC, HS‐A and cerebrovascular diseases, especially in MTL regions.

